# Supplementary material for: Increased vs. Standard Dose of Iron in Ready-to-Use Therapeutic Foods for the Treatment of Severe Acute Malnutrition in a Community Setting: A Systematic Review and Meta-Analysis
Source: Nutrients. 2022 Jul 29;14(15):3116. doi: 10.3390/nu14153116 (PMC9370784; doi:10.3390/nu14153116)
Supplement: Supplementary file 1 [file nutrients-14-03116-s001.zip › nutrients-1810087-supplementary.pdf]

# Annex S1: List of publications of included studies

| Title | Authors | Published Year | Merged Citation Title |
|---|---|---|---|
| Comparison of the effectiveness of a milk-free soy-maize-sorghum-based ready-to-use therapeutic food to standard ready-to-use therapeutic food with 25% milk in nutrition management of severely acutely malnourished Zambian children: an equivalence non-blinded cluster randomised controlled trial. | Irena, A. H., Bahwere, P., Owino, V. O., Diop, E. I., Bachmann, M. O., Mbwili-Muleya, C., Dibari, F., Sadler, K., & Collins, S. | 2015 | Owino VO, Irena AH, Dibari F, Collins S. Development and acceptability of a novel milk-free soybean-maize-sorghum ready-to-use therapeutic food (SMS-RUTF) based on industrial extrusion cooking process. *Matern Child Nutr*. Jan 2014;10(1):126-34. doi:10.1111/j.1740-8709.2012.00400.x<br><br>Irena AH, Bahwere P, Owino VO, et al. Comparison of the effectiveness of a milk-free soy-maize-sorghum-based ready-to-use therapeutic food to standard ready-to-use therapeutic food with 25% milk in nutrition management of severely acutely malnourished Zambian children: an equivalence non-blinded cluster randomised controlled trial. *Matern Child Nutr*. Dec 2015;11 Suppl 4:105-19. doi:10.1111/mcn.12054 |
| Cereals and pulse-based ready-to-use therapeutic food as an alternative to the standard milk- and peanut paste-based formulation for treating severe acute malnutrition: a noninferiority, individually randomized controlled efficacy clinical trial. | Bahwere, P., Balaluka, B., Wells, J. C., Mbiribindi, C. N., Sadler, K., Akomo, P., Dramaix-Wilmet, M., & Collins, S. | 2016 | |
| Soya, maize and sorghum ready-to-use therapeutic foods are more effective in correcting anaemia and iron deficiency than the standard ready-to-use therapeutic food: randomized controlled trial. | Akomo, P., Bahwere, P., Murakami, H., Banda, C., Maganga, E., Kathumba, S., Sadler, K., & Collins, S. | 2019 | Bahwere P, Akomo P, Mwale M, et al. Soya, maize, and sorghum-based ready-to-use therapeutic food with amino acid is as efficacious as the standard milk and peanut paste-based formulation for the treatment of severe acute malnutrition in children: a noninferiority individually randomized controlled efficacy clinical trial in Malawi. *Am J Clin Nutr*. Oct 2017;106(4):1100-1112. doi:10.3945/ajcn.117.156653<br><br>Akomo P, Bahwere P, Murakami H, et al. Soya, maize and sorghum ready-to-use therapeutic foods are more effective in correcting anaemia and iron deficiency than the standard ready-to-use therapeutic food: randomized controlled trial. *BMC Public Health*. Jun 24 2019;19(1):806. doi:10.1186/s12889-019-7170-x |

# Annex S2: List of excluded studies and reasons for exclusion

| Study | Reason for exclusion |
|---|---|
| Akomo 2020 (1) | Excluded for wrong study design as this publication was a commentary rather than a randomized trial. It commented on a trial that had investigated the micronutrient status of children before and after receiving RUTF, not on their hemoglobin, anemia status, weight, height, or other primary or secondary outcomes of this review. |
| Borg 2020 (2) | Excluded for wrong patient population as participants were healthy children and therefore did not meet inclusion criterion of diagnosis of severe acute malnutrition (SAM). |
| Delimont 2019 (3) | Excluded for wrong intervention as the study investigated the use of new formulations of fortified blended foods produced via extrusion compared to traditional preparations, not at differing amounts of iron content within ready-to-RUTF formulations. |
| Hieu 2012 (4) | Excluded for wrong patient population as participants did not meet the inclusion criterion of having a diagnosis of SAM. |
| Jayatissa 2012 (5) | Excluded for wrong study design as the study focused on the implementation and results of a nutrition rehabilitation program which was instituted for all children; there was no control group. |
| Maleta 2004 (6) | Excluded for wrong patient population as participants were moderately malnourished and therefore did not meet the inclusion criterion of having a diagnosis of SAM. |
| Nct 2010 (7) | Excluded for wrong intervention as the study investigated the use of supplementary iron administered separately, not as part of an RUTF formulation. |
| Nct 2016 (8) | Excluded for duplicate publication as this was a description of the clinical trial upon which Delimont 2019 study (see above) was based. |
| Olney 2017 (9) | Excluded for wrong patient population as participants did not meet inclusion criterion of diagnosis of SAM. Additionally, children as young as 0 months of age were included, and this analysis only included children aged 6 months or older. |
| Semba 2010 (10) | Excluded for wrong intervention as the study investigated the use of iron-fortified milk and noodles rather than ready-to-use therapeutic food. Additionally, the study was not a randomized trial. |
| Siega-Riz 2014 (11) | Excluded for wrong patient population as participants did not meet the inclusion criterion of having a diagnosis of SAM. |
| Sood 2002 (12) | Excluded for wrong intervention as participants in the experimental group were given an iron rich supplement but there was no control group being given a supplement with a different dose of iron. |
| van Stuijvenberg 1999 (13) | Excluded for wrong patient population as participants did not meet the inclusion criterion of having a diagnosis of SAM. |
| Kangas 2020 (14) | Excluded because the control group received less RUTF |

## Annex S3: Summary risk of bias for primary and secondary outcomes based on intention-to-treat

| Unique ID | Study ID | Experimental | Comparator | Outcome | Weight | D1 | D2 | D3 | D4 | D5 | Overall |
|---|---|---|---|---|---|---|---|---|---|---|---|
| 1 | 1 | High Iron Content | WHO iron content | Recovery rates | 1 | + | + | + | + | + | + |
| 2 | 2 | High Iron Content | WHO iron content | Recovery rates | 1 | + | + | + | + | + | + |
| 3 | 3 | High Iron content | WHO iron content | recovery rates | 1 | + | + | + | + | + | + |
| 4 | 4 | High Iron content | Low iron content | Blood hemoglobin | NA | + | + | - | + | + | - |
| 5 | 5 | NA | NA | Blood hemoglobin | 1 | + | + | - | + | + | - |
| 4 | 4 | High Iron content | Low iron content | Anemia | NA | + | + | - | + | + | - |
| 5 | 5 | NA | NA | Anemia | 1 | + | + | - | + | + | - |
| 4 | 4 | High Iron content | Low iron content | Severe anemia | NA | + | + | - | + | + | - |
| 5 | 5 | NA | NA | Severe anemia | 1 | + | + | - | + | + | - |
| 4 | 4 | High Iron content | Low iron content | Iron deficiency anemia | NA | + | + | - | + | + | - |
| 1 | 1 | High Iron Content | WHO iron content | All-cause mortality | 1 | + | + | + | + | + | + |
| 2 | 2 | High Iron Content | WHO iron content | All-cause mortality | 1 | + | + | + | + | + | + |
| 3 | 3 | High Iron content | WHO iron content | All-cause mortality | 1 | + | + | + | + | + | + |
| 1 | 1 | High Iron Content | WHO iron content | Default from trial | 1 | + | + | + | + | + | + |
| 2 | 2 | High Iron Content | WHO iron content | Default from trial | 1 | + | + | + | + | + | + |
| 1 | 1 | High Iron Content | WHO iron content | Default from trial | 1 | + | + | + | + | + | + |

| | |
|---|---|
| + | Low risk |
| ! | Some concerns |
| - | High risk |
| D1 | Randomisation proc |
| D2 | Deviations from the |
| D3 | Missing outcome da |
| D4 | Measurement of the |
| D5 | Selection of the rep |

**Footnotes:** The first three rows give risk of bias assessment for recovery rates for Bahwar 2016[16], Akomo2019 [15] and Irena 2015 [17]respectively. The next two rows give data on Blood Hb from Akomo 2019 and Bahware 2016 respectively. The row number 6 and 7 give risk of bias

assessment for anemia from the same two studies. Severe anemia was assessed for risk of bias in line 8 and 9 from the same studies. Iron deficiency anemia was reported in line 10 from Akomo 2019 only. All-cause mortality and default from trial was assessed in the last six rows in order of as Bahware 2016, Akomo 2019 and Irena 2015. The reason for high risk of bias from Akomo 2019 and Bahware 2016 studies was the data was reported for a subset of patients only.

# References


1. Akomo P, Collins S. Comment on RUTF and correction of anaemia and iron deficiency in severe acute malnutrition. Clin Nutr 2020;39:2935.
2. Borg B, Sok D, Mihrshahi S, et al. Effectiveness of a locally produced ready-to-use supplementary food in preventing growth faltering for children under 2 years in Cambodia: a cluster randomised controlled trial. Matern Child Nutr 2020;16:e12896.
3. Delimont NM, Vahl CI, Kayanda R, et al. Complementary Feeding of Sorghum-Based and Corn-Based Fortified Blended Foods Results in Similar Iron, Vitamin A, and Anthropometric Outcomes in the MFFAPP Tanzania Efficacy Study. Curr Dev Nutr 2019;3:nzz027.
4. Hieu NT, Sandalinas F, de Sesmaisons A, et al. Multi-micronutrient-fortified biscuits decreased the prevalence of anaemia and improved iron status, whereas weekly iron supplementation only improved iron status in Vietnamese school children. Br J Nutr 2012;108:1419-27.
5. Jayatissa R, Bekele A, Kethiswaran A, De Silva AH. Community-based management of severe and moderate acute malnutrition during emergencies in Sri Lanka: challenges of implementation. Food Nutr Bull 2012;33:251-60.
6. Maleta K, Kuittinen J, Duggan MB, et al. Supplementary feeding of underweight, stunted Malawian children with a ready-to-use food. J Pediatr Gastroenterol Nutr 2004;38:152-8.
7. Community-based Follow-up of Severely Malnourished Children. at https://ClinicalTrials.gov/show/NCT01157741.)
8. Newly Formulated, Extruded Fortified-blended Foods for Food Aid: the MFFAPP Tanzania Efficacy Study. at https://ClinicalTrials.gov/show/NCT02847962.)
9. Olney DK, Bliznashka L, Becquey E, Birba O, Ruel MT. Adding a Water, Sanitation and Hygiene Intervention and a Lipid-Based Nutrient Supplement to an Integrated Agriculture and Nutrition Program Improved the Nutritional Status of Young Burkinabé Children. The FASEB Journal 2017;31.
10. Semba RD, Moench-Pfanner R, Sun K, et al. Iron-fortified milk and noodle consumption is associated with lower risk of anemia among children aged 6-59 mo in Indonesia. Am J Clin Nutr 2010;92:170-6.
11. Siega-Riz AM, Estrada Del Campo Y, Kinlaw A, et al. Effect of supplementation with a lipid-based nutrient supplement on the micronutrient status of children aged 6-18 months living in the rural region of Intibuca, Honduras. Paediatr Perinat Epidemiol 2014;28:245-54.
12. Sood M, Sharada D. Iron food supplement. Indian J Pediatr 2002;69:943-6.
13. van Stuijvenberg ME, Kvalsvig JD, Faber M, Kruger M, Kenoyer DG, Benade AJ. Effect of iron-, iodine-, and beta-carotene-fortified biscuits on the micronutrient status of primary school children: a randomized controlled trial. Am J Clin Nutr 1999;69:497-503.
14. Kangas ST, Salpeteur C, Nikiema V, et al. Vitamin A and iron status of children before and after treatment of uncomplicated severe acute malnutrition. Clin Nutr 2020;39:3512-9.
15. Akomo, P.; Bahwere, P.; Murakami, H.; Banda, C.; Maganga, E.; Kathumba, S.; Sadler, K.; Collins, S. Soya, maize and sorghum ready-to-use therapeutic foods are more effective in correcting anaemia and iron deficiency than the standard ready-to-use therapeutic food: Randomized controlled trial. *BMC Public Health* **2019**, *19*, 806.
16 Bahwere, P.; Balaluka, B.; Wells, J.C.; Mbiribindi, C.N.; Sadler, K.; Akomo, P.; Dramaix-Wilmet, M.; Collins, S. Cereals and pulse-based ready-to-use therapeutic food as an alternative to the standard milk- and peanut paste-based formulation for treating severe acute malnutrition: A noninferiority, individually randomized controlled efficacy clinical trial. *Am. J. Clin. Nutr.* **2016**, *103*, 1145–1161.
17 Irena, A.H.; Bahwere, P.; Owino, V.O.; Diop, E.I.; Bachmann, M.O.; Mbwili-Muleya, C.; Dibari, F.; Sadler, K.; Collins, S. Comparison of the effectiveness of a milk-free soy-maize-sorghum-based ready-to-use therapeutic food to standard ready-to-use therapeutic food with 25% milk in nutrition management of severely acutely malnourished Zambian children: An equivalence non-blinded cluster randomised controlled trial. *Matern. Child Nutr.* **2015**, *11* (Suppl. 4), 105–119.
